# National Surveillance of Pediatric Acute Hepatitis of Unknown Etiology, Japan, October 2021–December 2022

**DOI:** 10.3201/eid2906.221579

**Published:** 2023-06

**Authors:** Shogo Otake, Chiaki Ikenoue, Natsu Sudani, Miho Kobayashi, Kensuke Takahashi, Tomoe Shimada, Itsuro Yoshimi, Tomoya Saito, Tomimasa Sunagawa

**Affiliations:** Kobe University Graduate School of Medicine, Kobe, Japan (S. Otake);; National Institute of Infectious Diseases, Tokyo, Japan (S. Otake, C. Ikenoue, N. Sudani, M. Kobayashi, K. Takahashi, T. Shimada, I. Yoshimi, T. Saito, T. Sunagawa)

**Keywords:** hepatitis, adenovirus infections, COVID-19, pediatrics, viruses, Japan, acute hepatitis of unknown etiology, AHUE

## Abstract

Pediatric acute hepatitis of unknown etiology has been reported globally since April 2022. In Japan, 139 possible cases with onset dates after October 2021 were reported as of December 2022. Three patients required liver transplants, but none died. Rates of adenovirus positivity (11/125, 9%) were lower than those for other countries.

Severe acute hepatitis of unknown etiology (AHUE) in children has been reported globally since April 2022. By July 8, 2022, a total of 1,010 cases had been reported to the World Health Organization from 35 countries on the basis of the working case definitions (*1*). A definition for a confirmed case is not available, but probable cases are defined as acute hepatitis (non-A–E hepatitis) in persons <16 years of age with serum transaminase >500 IU/L (aspartate transaminase or aspartate aminotransferase) since October 1, 2021; epidemiologically linked cases are acute hepatitis (non-A–E hepatitis) in persons of any age who were close contacts with a probable case-patient since October 1, 2021. Of the 1,010 cases identified, 46 (5%) children required liver transplants, and 22 (2%) children died (*1*). We report pediatric AHUE cases in Japan and compare them with cases in other countries. Because the data for this study were taken from an epidemiologic investigation conducted by the government, the National Institute of Infectious Diseases did not require informed consent and ethical review (receipt no. 1442).

The Ministry of Health, Labor and Welfare (MHLW) of Japan issued the working case definitions of AHUE on April 27, 2022 (*2*), adopting the case definition published by the World Health Organization but limiting cases to hospitalized patients (Appendix Table 1). Physicians were instructed to exclude viral hepatitis A, B, C, and E through laboratory tests and report cases to public health centers. Laboratories at hospitals and local public health institutions performed microbiological testing recommended by MHLW (Appendix Table 2). Acute liver failure was considered a coagulopathy characterized by a prothrombin time and international normalized ratio of >2 or >1.5 with clinical encephalopathy (*3*).

As of December 31, 2022, a total of 139 probable AHUE cases with onset dates after October 1, 2021, had been reported throughout Japan without geographic clustering (Table). Six cases with unknown onset dates were excluded, and none were epidemiologically linked. Among the 139 patients, 3 (2%) underwent liver transplantation. Eleven (13%) of 85 patients met the definition of acute liver failure, 17 (18%) of 95 received intensive care, and none died (Table).

**Table:** Characteristics and laboratory findings of 139 cases that fulfilled the working case definition of pediatric acute hepatitis of unknown etiology, Japan, October 2021–December 2022*

Characteristic	Value
Median age, y (IQR)	4.4 (1.3–9.5)
<6 y of age	81/139 (58)
Sex	
M	74/139 (53)
F	65/139 (47)
Any comorbidities†	37/139 (26)
No comorbidities	98/139 (71)
Presence of comorbidities unknown	4/139 (3)
History of COVID-19 before onset of disease	15/132 (11)
Median duration from COVID-19 onset to hepatitis onset, d (range)	85 (14–300)
Persons >5 y who received >1 dose of COVID-19 vaccine	22/66 (33)
Any international travel in 2 mo before illness	0/130 (0)
Any contact with sick persons in 2 wk before illness	39/129 (30)
Treatment	
Steroid therapy	15/139 (11)
Immunoglobulin	6/139 (4)
Plasmapheresis	6/139 (4)
Hemodialysis	4/139 (3)
Liver transplantation	3/139 (2)
Outcome	
Acute liver failure	11/85 (13)‡
Hospitalized to ICU or HCU	17/95 (18)
Death	0/139 (0)
Median duration from symptom onset to hospital admission, d (IQR)	4 (2–7.5)
Median length of hospital stay, d (IQR)	10 (7–16)
Clinical symptoms§	
Fever 37.5°C or higher	89/138 (64)
Gastrointestinal symptoms: abdominal pain, diarrhea, or nausea/vomiting	75/138 (54)
Cough	29/138 (21)
Jaundice	29/138 (21)
White stools	10/138 (7)
Impaired consciousness	6/138 (4)
Median AST, IU/L (IQR)¶	764 (503–1,312)
Median ALT, IU/L (IQR)¶	838 (576–1,390)
Median total bilirubin, mg/dL (IQR)¶	1.00 (0.60–4.74)
Median PT-INR (IQR)¶	1.11 (1.02–1.32)
No. SARS-CoV-2 positive/no. tested (%)	10/134 (7)
Nucleic acid amplification test: PCR 101, LAMP 1, and NEAR 1	8/103 (8)
Antigen test	2/13 (15)
Type of test unknown	0/18 (0)

*Values are no. (%) except as indicated. Denominators consist of cases for which data are available. ALT, alanine aminotransferase; AST, aspartate aminotransferase; HCU, high-care unit; ICU, intensive care unit; IQR, interquartile range; LAMP, loop-mediated Isothermal amplification; NEAR, nicking enzyme amplification reaction; PT-INR, prothrombin time and international normalized ratio. †Specific underlying conditions reported were psychomotor retardation (11, 8%), syndromes involving changes in chromosomes or genes (5, 4%), congenital heart disease (4, 3%), congenital metabolic disorder (3, 2%), low birthweight (3, 2%), endocrine disorder (3, 2%), autoimmune and collagen diseases (3, 2%), primary immunodeficiency syndrome (2, 1%), and other disorders (8, 6%) (atopic dermatitis, cloacal exstrophy, hydronephrosis, unilateral kidney agenesis and haemangioma). ‡Including 3 patients with encephalopathy. §Some patients reported >1 sign/symptom. ¶Maximum values up to the time of reporting. Based on information from 136 (AST and ALT), 99 (total bilirubin), and 85 (PT-INR) cases.

Of note, of 125 cases tested for adenovirus by PCR, 11 (9%) were positive (Appendix Table 3); however, adenoviruses were the most frequently detected pathogen in AHUE cases from Europe (52%) and the United Kingdom (66%) (*4*,^5^). Among the 11 adenovirus-positive cases, type 41 was identified in only 2 cases (18%) in Japan, unlike its frequent detection in England (*5*) (Appendix Table 1). Studies from the United Kingdom reported simultaneous increases in numbers of hospitalized hepatitis case-patients and detected adenoviruses cases (*5*). In Japan, the national surveillance system for viral hepatitis (Appendix Table 4), adenovirus, and adenovirus-associated syndromes (e.g., pharyngoconjunctival fever) did not identify unusual numbers or trends compared with previous years (*2*). The varying characteristics of reported AHUE cases among countries might be attributed to these differences.

Some reports have stated that SARS-CoV-2 spike protein acts as a superantigen, broadly stimulating T cells to induce hyperinflammation and potentially contributing to hepatitis (*6*). AHUE cases in Europe and United Kingdom revealed high rates of SARS-CoV-2 seropositivity (*4*,*5*) (Appendix Table 1). However, our study indicated low SARS-CoV-2 positivity (10/134, 7%) at the time of hospitalization for AHUE in Japan. Results of serologic tests for SARS-CoV-2 were unavailable because they were not required. The low proportion of patients with a history of COVID-19 before onset of AHUE (15/132, 11%) might explain the lower rates of seropositivity in Japan than for Europe and the United Kingdom.

Laboratory tests did not reveal a high frequency of any specific microorganism in Japan, and the distribution, other than for adenovirus, was similar to that reported in Europe (*4*). The cause of AHUE in Japan remains unknown. Cases reported in Japan were less severe than those reported in other countries (*1*,*2*,*4*,*5*,*7*) (Appendix Table 1), which might be because of differences in genetic predisposition that could affect inflammatory responses and clinical severity, as has been suggested with certain acute inflammatory diseases (*8*). The prevalence of the HLA-DRB1*04:01 allele, expressed by 89% of AHUE liver transplant cases in Scotland (*5*), is higher in the general population in Scotland than in Japan (8.9% vs. 1.0%) (*9*).

The first limitation of our study is that ascertainment bias might have affected microbiological testing results. The pathogens listed by MHLW (Appendix Table 2) might not have been examined uniformly and systematically, and the frequency of pathogens indicated in this report might not accurately reflect actual distribution. Second, the increase in reports after MHLW issued an administrative notice could be caused by reporting bias (Figure). Last, recall bias could have resulted in underestimates of the number of AHUE cases early in the study period. 

**Figure:**
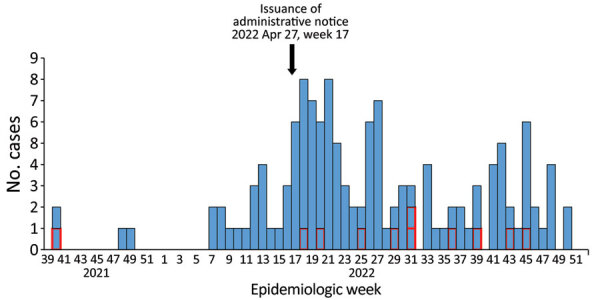
Cases of acute hepatitis of unknown etiology by week of onset in Japan, October 2021– December 31, 2022. The Ministry of Health, Labour, and Welfare Japan issued the working case definitions and administrative notice on April 27, 2022. In total, 139 probable cases with onset dates after October 1, 2021 (week 39, 2021), were reported as of December 31, 2022 (week 52, 2022). We excluded 6 cases for which onset dates were unavailable. Red outlines indicate cases fulfilling the diagnostic criteria for acute liver failure (n = 11).

In conclusion, 23 identified 139 pediatric AHUE cases in Japan during October 2021–December 2022 that differed in severity and adenovirus PCR positivity from cases in other countries. However, no unusual trends were found in this investigation. Japan might observe similar AHUE trends as in past years, as in the United States (*10*). 

Appendix Additional information about national surveillance of pediatric acute hepatitis of unknown etiology, Japan, October 2021–December 2022
