# Supplementary material for: National Surveillance of Pediatric Acute Hepatitis of Unknown Etiology, Japan, October 2021–December 2022
Source: Emerg Infect Dis. 2023 Jun;29(6):1288–91. doi: 10.3201/eid2906.221579 (PMC10202856; doi:10.3201/eid2906.221579)
Supplement: Appendix — Additional information about national surveillance of pediatric acute hepatitis of unknown etiology, Japan, October 2021–December 2022 [file 22-1579-Techapp-s1.pdf]

Article DOI: https://doi.org/10.3201/eid2906.221579

EID cannot ensure accessibility for supplementary materials supplied by authors. Readers who have difficulty accessing supplementary content should contact the authors for assistance.

National Surveillance of Pediatric Acute Hepatitis of Unknown Etiology, Japan, October 2021–December 2022

Appendix

Appendix Table 1. Case definition and characteristics of pediatric acute hepatitis of unknown etiology in each country/region

Probable case definition published by WHO (*1*)	Person presenting with acute hepatitis (non-hepatitis A-E) with serum transaminase >500 IU/L (AST or ALT) who is 16 y old or younger, since 1 October 2021			
Country/Region	Japan (*2*)	EU/EEA (*3*)	U.S. (*4*)	UK (*5*)
Differences from WHO probable case definition	Probable case includes only hospitalized cases and excludes acute hepatitis with identifiable cause.	Probable case excludes cases of hepatitis with known etiology such those due to specific infectious diseases, drug toxicity, metabolic hereditary, or autoimmune disorders.	<10 y old	・15 y old or younger (0–10 y: confirmed case, 11–15 y: possible case)<br>・Excluding metabolic, inherited, or genetic, congenital, or mechanical cause.<br>・Scotland case definition also exclude cases due to cytomegalovirus, or Epstein-Barr Virus.
Investigation period	October 1, 2021-December 31, 2022	October 1, 2021-November 24,2022	October 1, 2021-June 15, 2022	January 1, 2022-July 4, 2022
Number of reported cases	139 (Probable cases)	572 (Probable cases)	296	274 (Confirmed: 263, Possible: 11)
Acute liver failure	13% (11/85)	-	30% (37/123)	-

Probable case definition published by WHO (*1*)	Person presenting with acute hepatitis (non-hepatitis A-E) with serum transaminase >500 IU/L (AST or ALT) who is 16 y old or younger, since 1 October 2021			
Hospitalized to ICU or HCU	18% (17/95)	27% (100/371)	-	-
Liver transplantation	2% (3/139)	8% (24/320)	6% (18/296)	5% (15/274)
Death	0% (0/139)	2% (7/405)	4% (11/296)	0% (0/274)
Adenovirus test positivity	9% (11/125)	52% (236/457)	45% (100/224)	66% (170/258)
Type 41	18% (2/11)	42% (5/12)	46% (6/13)	92% (48/52)*
SARS-CoV-2 test positivity	7% (10/134)	10% (40/392)	10% (10/98)	15% (36/237)
SARS-CoV-2 serology test positivity	-	64% (73/115)	-	61% (1- to 4-y-olds), 67% (5- to 10-y-olds) †

ALT, alanine aminotransferase; AST, aspartate aminotransferase; EU/EEA, European Union and European Economic Area; HCU, high care unit; ICU, intensive care unit; UK, United Kingdom; U.S., United States; WHO, World Health Organization

* Data in England

† The details of the numerator and denominator are unknown.

Appendix Table 2. Recommended testing lists in medical institution and local public health institutions by the Ministry of Health, Labor and Welfare

Testing recommendations	
For medical institution	
Virus	Recommended
HAV	Anti-HAV antibody (IgM)
HBV	Hepatitis B surface antigen, anti-HBc antibody
HCV	Anti-HCV antibody
HEV	Anti-HEV antibody (IgA or IgM)
CMV	Anti -CMV antibody (IgM), CMV antigen, CMV PCR test
EBV	Anti-VCA antibody (IgM or IgG), EBV-nuclear antigen antibody
HSV	HSV-1,2 PCR test
SARS-CoV-2	SARS-CoV-2 PCR
For local public health institutions	
Test for adenovirus is recommended first and, the type should be determined, if test is positive.	
Type of sample	Recommended test (PCR test or bacterial culture)
Blood	Enterovirus*, Parechovirus†, HSV-1, 2, CMV, VZV, EBV, HHV-6, 7
Stool	Enterovirus*, Sapovirus, Norovirus, Rotavirus, *Salmonella* spp., *Shigella* spp., *Campylobacter* spp., Enteropathogenic *Escherichia coli*

Testing recommendations	
Respiratory sample	Enterovirus*, Influenza virus, SARS-CoV-2

CMV, cytomegalovirus; EBV, Epstein-Barr virus; HAV, hepatitis A virus; HBV, hepatitis B virus; HCV, hepatitis C virus; HEV, hepatitis E virus; HHV, human herpes virus; HSV, Herpes simplex virus; IgA, immunoglobulin A; IgG, immunoglobulin G; IgM, immunoglobulin M; PCR, polymerase chain reaction; VCA, viral capsid antigen; VZV, Varicella zoster virus

* If positive, the type should be determined.

† Test should be considered according to age.

Appendix Table 3. Laboratory findings for 139 cases that fulfilled the working case definition of pediatric acute hepatitis of unknown etiology, Japan, October 2021–December 2022*

	No. positive/no. tested (%)					
Viruses detected by PCR	Any specimen	Whole blood/plasma	Serum	Respiratory	Stool	Urine
Adenovirus	11/125 (9)†	1/91 (1)	3/93 (3)	6/101 (6)	7/95 (7)	2/56 (4)
Rhinovirus/enterovirus‡	14/86 (16)	0/53 (0)	0/59 (0)	14/71 (20)	3/56 (5)	1/31 (3)
Human herpes virus 6	4/44 (9)	1/29 (3)	2/31 (6)	1/23 (4)	0/17 (0)	0/13 (0)
Human herpes virus 7	4/41 (10)	2/27 (7)	1/31 (3)	2/23 (9)	0/17 (0)	0/13 (0)
Epstein-Barr virus	4/36 (11)	1/23 (4)	0/22 (0)	2/18 (11)	2/13 (15)	0/11 (0)
Norovirus	3/53 (6)	NT	NT	NT	3/53 (6)	NT
Cytomegalovirus	2/42 (5)	2/29 (7)	1/28 (4)	1/19 (5)	1/14 (7)	0/12 (0)
Herpes simplex virus 1	2/51 (4)	0/36 (0)	0/31 (0)	2/20 (10)	0/15 (0)	0/12 (0)
Human parechovirus 3	1/37 (3)	0/28 (0)	0/28 (0)	1/25 (4)	1/27 (4)	0/16 (0)
Human parainfluenza 3	1/49 (2)	0/21 (0)	1/25 (4)	1/44 (2)	0/20 (0)	0/13 (0)
Rotavirus	1/52 (2)	NT	NT	NT	1/52 (2)	NT
Sapovirus	1/49 (2)	NT	NT	NT	1/49 (2)	NT

*NT, not tested.

†There were 2 cases of type 41 and 1 case each of adenovirus type 1, type 2, type 3, and type 1 and 2, and 5 cases of unknown serotype.

‡Because the PCR tests in some cases could not distinguish between rhinovirus and enterovirus, we integrated them.

Appendix Table 4. Notification criteria of viral hepatitis based on the national law (Act on the Prevention of Infectious Diseases and Medical Care for Patients with Infectious Diseases)

Case definition	An acute hepatitis caused by hepatitis A-E virus or other viruses including asymptomatic cases. Chronic liver disease, asymptomatic carriers, and acute-on-chronic liver failure due to hepatitis B and C should be excluded.
Notification criteria	Physicians must notify the prefectural governor if they diagnose viral hepatitis based on clinical symptoms and laboratory findings such as serology test or polymerase chain reaction test, regardless of severity.

References

1. World Health Organization. Severe acute hepatitis of unknown etiology in children—multi-country. 2022 Jul 12 [cited 2022 Oct 15]. https://www.who.int/emergencies/disease-outbreak-news/item/2022-DON400

2. National Institute of Infectious Diseases. Acute hepatitis of unknown etiology in children in Japan, October 2021–June 2022, as at Jun 23, 2022 (First report). 2022 Jul 27 [cited 2022 Oct 15]. https://www.niid.go.jp/niid/en/all-surveillance/2603-fetp/jissekijpn/11344-acute-hepatitis-of-unknown-etiology-in-children-in-japan-october-2021-june-2022-as-at-23-june-2022-first-report.html

3. European Centre for Disease Prevention and Control. Joint ECDC-WHO regional office for Europe hepatitis of unknown origin in children surveillance bulletin. 2022 Nov 25 [cited 2022 Dec 31]. https://www.ecdc.europa.eu/en/hepatitis/joint-hepatitis-unknown-origin-children-surveillance-bulletin

4. Cates J, Baker JM, Almendares O, Kambhampati AK, Burke RM, Balachandran N, et al.; Hepatitis of Unknown Etiology Group. Interim analysis of acute hepatitis of unknown etiology in children aged <10 years–United States, October 2021–June 2022. MMWR Morb Mortal Wkly Rep. 2022;71:852–8. PubMed https://doi.org/10.15585/mmwr.mm7126e1

5. UK Health Security Agency. Investigation into acute hepatitis of unknown etiology in children in England. Technical briefing 4. 2022 Jul 26 [cited 2022 Oct 15]. https://assets.publishing.service.gov.uk/government/uploads/system/uploads/attachment_data/file/1094573/acute-hepatitis-technical-briefing-4.pdf
